# Localization accuracy of multiple magnets in a myokinetic control interface

**DOI:** 10.1038/s41598-021-84390-8

**Published:** 2021-03-01

**Authors:** Marta Gherardini, Francesco Clemente, Stefano Milici, Christian Cipriani

**Affiliations:** 1 The Biorobotics Institute, Scuola Superiore Sant’Anna, 56127 Pisa, Italy; 2 Department of Excellence in Robotics & AI, Scuola Superiore Sant’Anna, 56127 Pisa, Italy

**Keywords:** Engineering, Biomedical engineering

## Abstract

Magnetic localizers have been widely investigated in the biomedical field, especially for intra-body applications, because they don’t require a free line-of-sight between the implanted magnets and the magnetic field sensors. However, while researchers have focused on narrow and specific aspects of the localization problem, no one has comprehensively searched for general design rules for accurately localizing multiple magnetic objectives. In this study, we sought to systematically analyse the effects of remanent magnetization, number of sensors, and geometrical configuration (i.e. distance among magnets—L_inter-MM_—and between magnets and sensors—L_MM-sensor_) on the accuracy of the localizer in order to unveil the basic principles of the localization problem. Specifically, through simulations validated with a physical system, we observed that the accuracy of the localization was mainly affected by a specific angle ($\theta$ = tan^−1^(L_inter-MM_ / L_MM-sensor_)), descriptive of the system geometry. In particular, while tracking nine magnets, errors below ~ 1 mm (10% of the length of the simulated trajectory) and around 9° were obtained if θ ≥  ~ 31°. The latter proved a general rule across all tested conditions, also when the number of magnets was doubled. Our results are interesting for a whole range of biomedical engineering applications exploiting multiple-magnets tracking, such as human–machine interfaces, capsule endoscopy, ventriculostomy interventions, and endovascular catheter navigation.

## Introduction

Magnetic tracking systems have been widely investigated because of their important applications in the biomedical engineering field. Indeed, the transparency of the human body to low-frequency magnetic fields, and the fact that a free line-of-sight between the magnetic markers (MMs) and the tracker is not needed^1^, make such systems highly suitable for intra-body applications^2^. As an example, magnetic tracking systems can be exploited for guiding and dragging endoscopic capsules inside the gastrointestinal tract^3–5^, improving the guidance of ventriculostomy interventions^6^, as well as navigating catheters during cardiovascular procedures^7^. Another potential application was recently featured by our group, which foresees to exploit a magnetic tracking system for controlling prosthetic limbs, e.g. a prosthetic hand^8^ (the *myokinetic control interface*). In particular, by tracking the displacement of passive MMs (i.e., permanent magnets) implanted in the forearm muscles following contraction, we could exploit this information to control the relative degrees of freedom (DoFs) in the prosthesis.

Although in the literature the tracking is often limited to a single magnet^9–11^, few examples of multi-objectives localization include the trackers developed by Yang et al.^12^, Taylor et al.^13^ and Tarantino et al.^14^, which are capable of simultaneously localize up to three (15 DoFs), four (20 DoFs) and seven MMs (35 DoFs), respectively. Several applications, including the one proposed by our group, would highly benefit from an increase in the number of simultaneously trackable MMs^8,12,15^. In our first work, we proved the viability of a system able to localize the position of four MMs ^8^ *virtually implanted* in an anatomically relevant forearm mockup; in a more recent study^16^, we demonstrated an embedded system capable of localizing up to five MMs, in real-time. Besides the practical implementation of the system, we investigated how the accuracy, the precision and the computation time of the localizer are affected by the number and distribution of both the MMs and the sensors^14^. To this aim, up to nine MMs were simulated using Finite Element Modelling and moved along random trajectories inside an anatomically relevant workspace. The system proved able to localize up to seven MMs with good accuracy, while the configurations with nine MMs showed much larger errors than all other configurations. In addition, the localization accuracy proved negatively correlated with L_MM-sensor_ (defined as the distance between the magnets and the sensors), corroborating previous findings^6,9,17,18^, and positively correlated with L_inter-MM_ (defined as the inter-magnet distance).

Building on this, in this study we sought to systematically analyse the effects of remanent magnetization (*B*_*r*_), number of sensors, L_inter-MM_ and L_MM-sensor_ on the accuracy of the localizer, in order to unveil some of the basic principles of the localization problem or, in other words, define effective guidelines (or design tips) for optimal myokinetic controllers. Indeed, thus far, while researchers have focused on narrow and specific aspects of the problem, no one has comprehensively searched for such general design rules for multiple magnetic objectives. As an example, Hu et al. studied the effects of the number and the arrangement of sensors on the faces of a cube, on the localization accuracy of a single magnet^17^. Talcott analytically investigated the optimal sensor layout on a surface, based on the position and orientation of one magnetic target^19^. Following a different approach, Schlageter and colleagues used a 2D array of sensors to analyse the effect on the localization of the position and orientation of a permanent magnet, along with that of different error sources^18^. All studies confirmed that the localization accuracy correlates positively with the number of sensors and negatively with L_MM-sensor_. Moreover, results from^18,19^ highlighted that a better accuracy can be achieved when the sensors are aligned with the magnetization axis of the magnet, as the signal to noise ratio is increased. However, generalizing these outcomes from a single to multiple magnets (and all that it entails) represents a fundamental step towards the understanding of the underlying phenomena and the development of a new class of magnetic localizers.

**Figure 1:**
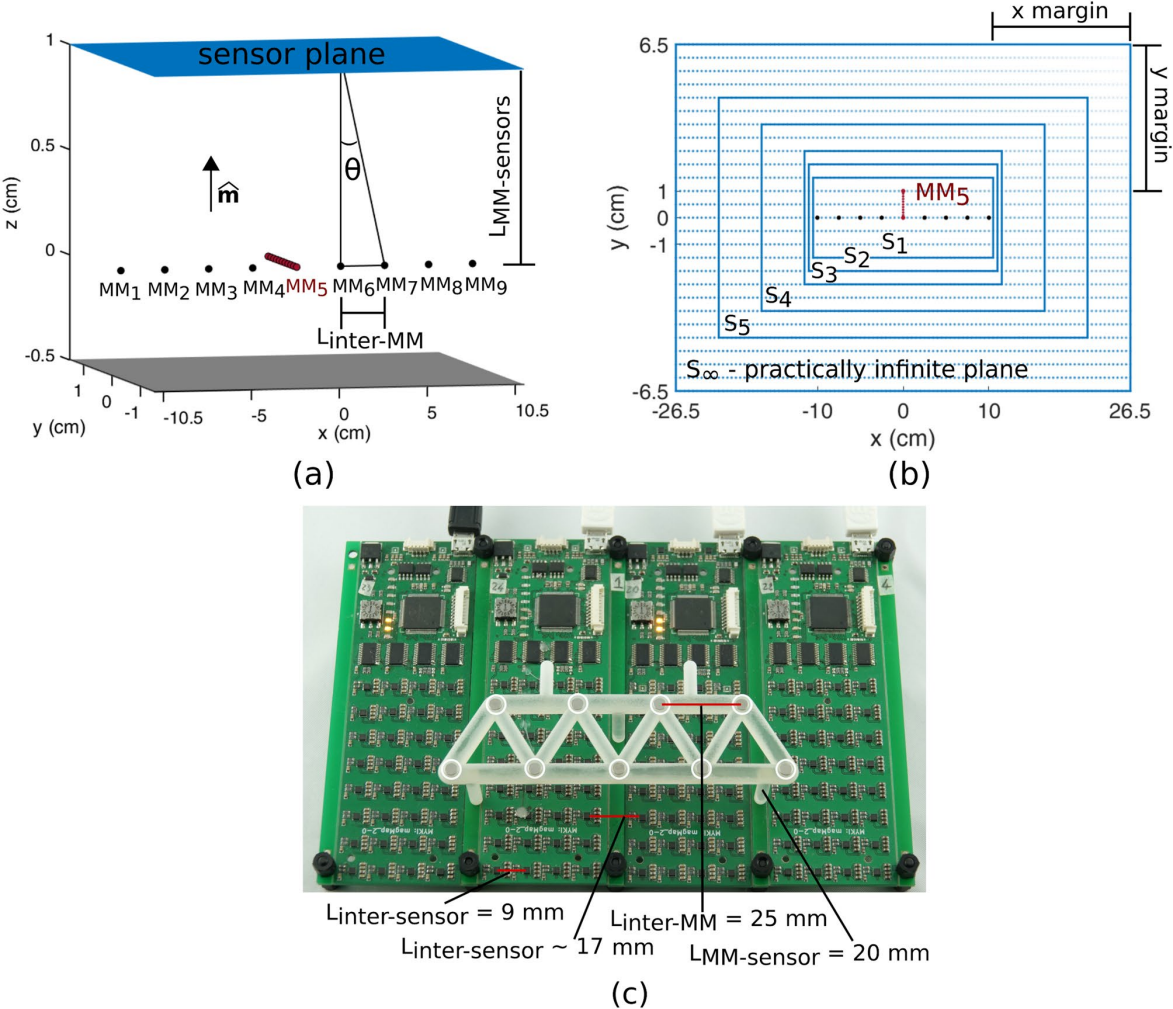
Simulated and physical setups. (**a**) Typical simulated setup, with nine equidistant (L_inter-MM_) and aligned magnets (MM) at a distance L_MM-sensor_ from a sensor surface/plane. A movement of the central MM (i.e., MM_5_) along a 10 mm trajectory parallel to the sensor plane was simulated. Different geometrical configurations (i.e. different L_MM-sensor_ and L_inter-MM_) characterized by the same angle θ were considered as equivalent and clustered together. The black arrow indicates the magnetization vectors of the MMs. (**b**) Different sensor planes (S_∞_, S_5_–S_1_) were simulated by varying the distance (x margin and y margin) between the external magnets and the edge of the plane along both the x (x margin) and the y (y margin) axis. (**c**) Physical system used in the control test. Four custom boards including 32 sensors each (for a total of 128 sensors) were used to sample the magnetic field generated by nine MMs (identified by the white circles). The latter were held (at 20 mm from the sensors plane) in a static configuration using a 3D-printed frame featuring a zigzag pattern.

Thus, taking our previous study as a starting point^14^, here we simulated different setups in which the number of MMs was fixed at nine, while their remanent magnetization *B*_*r*_, L_MM-sensor_, L_inter-MM_ and the number of sensors were varied. Both the sensors and the magnets were placed on a planar configuration, being this a simplification of more complex setups (e.g. the circular geometry of the human forearm) (Fig. 1a). We first considered a practically infinite sensing surface and then progressively reduced it, in order to investigate its influence on the localization error (Fig. 1b). Markers were aligned on a plane parallel to the sensors, and only the one in the central position was moved along a linear trajectory of 10 mm. For each setup, we assessed the localization errors of the permanent magnets both in terms of position and orientation (namely, the pose). We observed that the accuracy of the localization was unaffected by the magnetization of the MMs but just by a specific angle, which we will refer to as θ, that depends on the ratio between L_MM-sensor_ and L_inter-MM_ (Fig. 1a), and is thus descriptive of the geometry of the system (Fig. 1a). In fact errors below ~ 1 mm were obtained when θ was equal or higher than ~ 31°. The latter proved a general rule across a wide spectrum of conditions, and when doubling the number of simulated MMs or in control tests employing a physical demonstrator (Fig. 1c). In addition, θ greater than ~ 31° also ensured an orientation error around 9°, and always below 15°.

These outcomes are of interest for a range of applications in the engineering and the biomedical fields in which tracking of multiple MMs or bodies is required. As an example, in the case of a myokinetic control interface, they represent an important step forward towards the development of a highly intuitive and physiologically appropriate controller for limb prostheses.

## Results

Six different sensing areas were simulated (i.e., *S*_*∞*_*, S*_*5*_*-S*_*1*_), in which the number of sensors was reduced from 2889 (*S*_*∞*_—practically infinite plane) to 301 (*S*_*1*_) (Fig. 1b, Table 1). L_inter-MM_ ranged between 15 and 25 mm, in steps of 5 mm, while L_MM-sensor_ ranged between 10 and 40 mm, in steps of 10 mm. Each possible combination of such distances was simulated for all the sensing areas and for three different magnet grades (namely, N35, N45 and N52, associated with an increasing *B*_*r*_). Akin to our previous work^14^, magnets were modelled as point magnetic dipoles (see Eq. (1) in Materials and Methods) and their position was retrieved based on the magnetic field readings through an iterative optimization procedure (Levenberg–Marquardt optimization method). Additionally, the accuracy of the localization was evaluated (conservatively) as the 95th percentile of the model error (${e}_{m}$) and the cross-talk error (${e}_{ct}$), for both position and orientation^8^. As in our previous work^8^, the former is the localization error measured for the moving magnet, while the latter is the error measured for the non-moving magnets (representing the false prediction of simultaneous displacements). Results obtained in different conditions were clustered according to the θ angle (Fig. 1a).

**Table 1:** Sensor planes size and number of sensors.

Setup	# Sensors per row*	# Sensors per column*	x margin (mm)^†^	y margin (mm)^†^	Sensing surface area (mm^2^)
S_∞_	107	27	165	55	689 × 10^2^
S_5_	87	19	115	35	387 × 10^2^
S_4_	67	15	65	25	231 × 10^2^
S_3_	47	11	15	15	115 × 10^2^
S_2_	45	9	10	10	88 × 10^2^
S_1_	43	7	05	05	63 × 10^2^

*The number of sensors per row (x axis) and per column (y axis) is reported.

^†^The x margin refers to the minimum distance between the edge of the sensor surface and the centre of the external magnet along the x direction (i.e., when the maximum L_inter-MM_ of 25 mm is considered); the y margin represents the distance between the edge of the sensor surface and the moving magnet (i.e., MM_5_) in correspondence of its maximum stroke of 10 mm**.**

### Simulated setup

**Figure 2:**
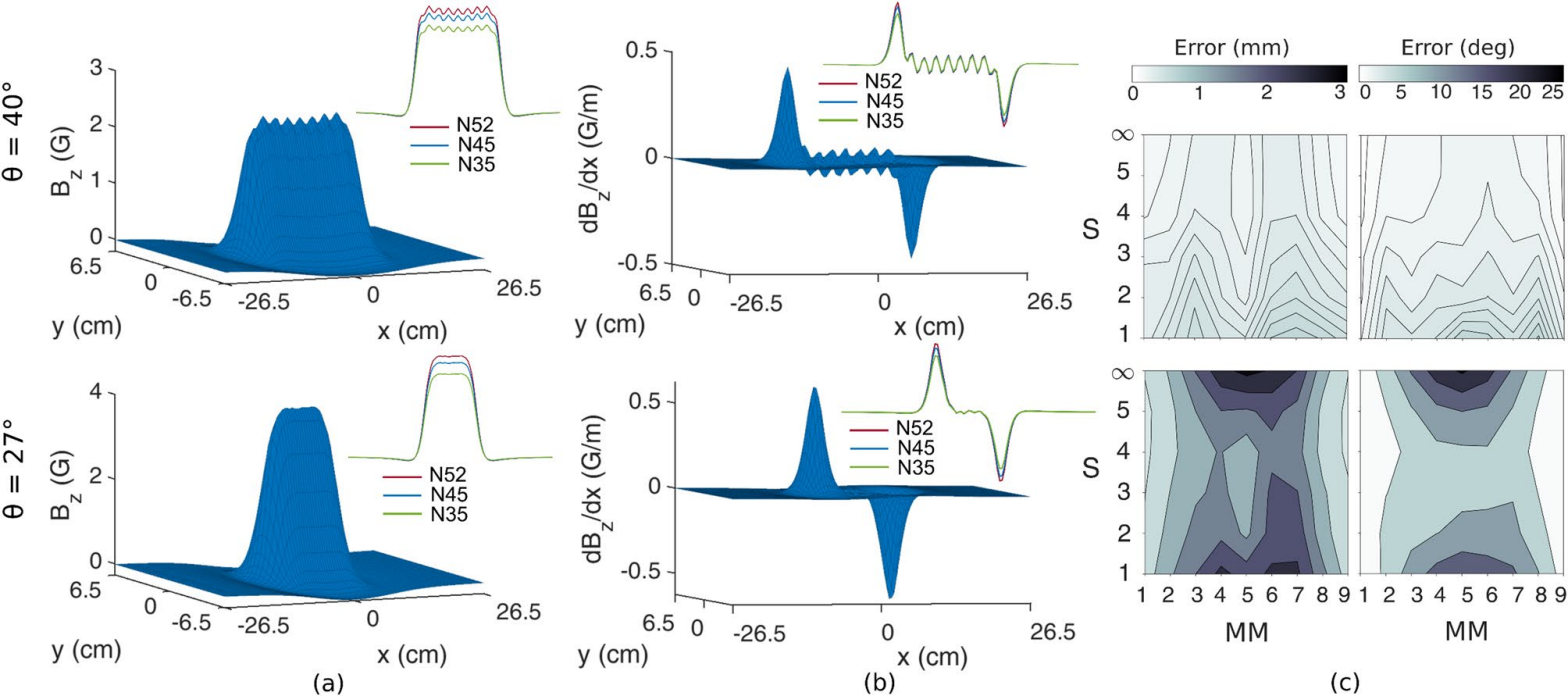
Results from two representative configurations (θ = 40°, upper panels, and θ = 27°, lower panels—N45 grade magnets). (**a**) Spatial distribution of the magnetic field z-component (B_z_) measured by the sensor plane (S_∞_); insets: B_z_ for all magnet grades measured for y = 0 cm. The magnetic field appears as the linear combination of bell-shaped waveforms, each one generated by one magnet. For θ = 40°, the peaks of the bells are always clearly distinguishable, while the ripple becomes less evident for θ = 27°. (**b**) B_z_ gradient (along x) on S_∞_; insets: Bz gradient (along x) for all magnet grades measured for y = 0 cm. Peaks corresponding to different magnets proved more (less) evident for θ = 40° (27°). (**c**) Contour plots displaying position (on the left) and orientation (on the right) ${e}_{m}$ and ${e}_{ct}$, for all sensing areas and all MMs (95th percentile). Good accuracies were achieved for θ = 40°, vice-versa errors increased for θ = 27°. In both cases, ${e}_{ct}$ decreased when moving from the central towards external MMs.

The magnetic field produced by a number of magnets in a row appears as the linear combination of bell-shaped waveforms, each one caused by one magnet (Fig. 2a). As an example, for N45 grade magnets and θ equal to 40°, the peaks of the bells were clearly distinguishable, from both the spatial distribution of the field and its gradient (Fig. 2a,b). For θ = 27° instead, the ripple became less evident and the magnetic field appeared as spatially smoothened. In other words, the peaks/magnets proved more or less evident based on the geometrical configuration described by θ, being more distinguishable when the magnets were far enough from each other or the distance to the sensors was sufficiently small. This held true for all the tested magnet grades (insets in Fig. 2a,b). On the contrary, the peaks associated to the external magnets were in all cases clearly visible from the gradient of the magnetic field (Fig. 2b).

The differences in the magnetic field shapes due to the geometrical factors corresponded to different degrees of accuracy of the solution of the localization problem (Fig. 2c). For N45 grade magnets and θ equal to 40°, the ${e}_{m}$ and ${e}_{ct}$ proved always lower than 0.59 mm and 5.12°, exhibiting a positive correlation with the reduction of the sensing area (Fig. 2c, upper panel). In addition the ${e}_{ct}$ demonstrated a decreasing trend when moving from the central magnets to more external ones; in fact, the MMs at the edges of the distribution (i.e., MM_1_ and MM_9_) exhibited the lowest error. In the representative case with N45 grade magnets and θ = 27°, ${e}_{m}$ and ${e}_{ct}$ proved always greater (roughly quadruplicated or more) than the corresponding cases with θ = 40° (Fig. 2c, lower panel). However, while the decreasing trend of ${e}_{ct}$ with the distance to the moving magnet was confirmed, the correlation between localization errors and sensing area did not appear clear as with θ = 40°.

**Figure 3:**
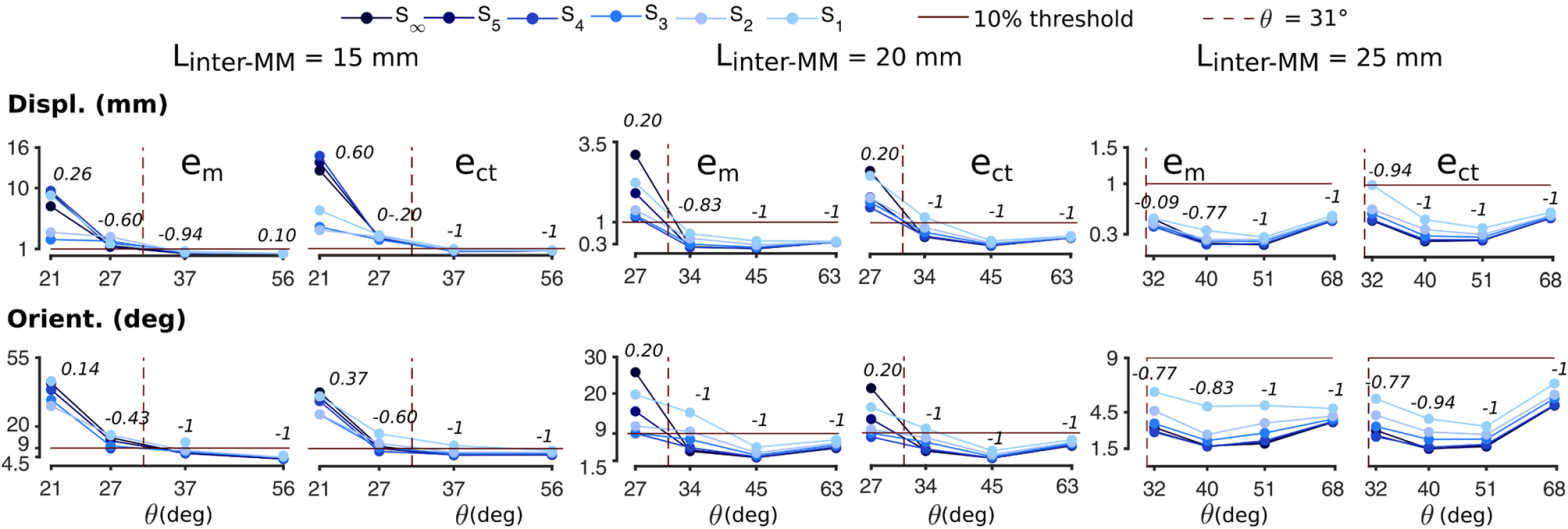
Displacement errors in all tested configurations for the N45 grade magnets as a function of θ. The 10% threshold (red solid line) corresponds to 10% the MM_5_ trajectory length (i.e. 1 mm) and the right angle. For θ ≥ 31° (threshold identified by the red dashed line), errors (95th percentile) proved generally below the 10% threshold, and showed a negative correlation with the sensing area (numbers in italics indicate the ρ_s_ value for each θ). Such correlation was lost for θ < 31°, where errors proved always above the 10% threshold. Both position (first row) and orientation (second row) errors are shown.

The results from the two representative cases for N45 grade magnets (θ = 27° and 40° in Fig. 2) nicely generalized across the spectrum of L_inter-MM_ and L_MM-sensor_ combinations (Fig. 3). Specifically, ${e}_{m}$ and ${e}_{ct}$ proved almost always negatively correlated with L_MM-sensor_ (i.e., negatively correlated with θ) for each given value of L_inter-MM_, and—in absolute terms—negatively correlated with L_inter-MM_. For example, if we consider L_inter-MM_ equal to 15 mm and *S*_*∞*_, the position ${e}_{m}$ decreased from 7.32 mm (for θ = 21°) to 0.34 mm (for 56°); for L_inter-MM_ equal to 25 mm and *S*_*∞*_, the ${e}_{m}$ decreased from 0.51 mm (for θ = 32°) to 0.15 mm (θ = 51°), increasing again to 0.49 mm (for θ = 68°) (Fig. 3). With respect to the surface area, all the displacement errors (both in position and orientation) exhibited different trends based on θ, in a very consistent manner across conditions. In fact, for θ ≥ 31°, the errors generally proved negatively correlated with the decrease of the sensing area (i.e., from *S*_*∞*_ to *S*_*1*_). In particular, the Spearman correlation coefficient (ρ_s_) between each of ${e}_{m}$, ${e}_{ct}$ and θ, and the decreasing sensing areas proved always between − 1 and − 0.77, except for two cases (namely, the position error ${e}_{m}$ for θ = 68° / L_inter-MM_ = 15 mm, and θ = 32° / L_inter-MM_ = 25 mm) (Fig. 3). Vice-versa, for θ < 31°, this same correlation was lost, as the ρ_s_ ranged between − 0.60 and 0.14 (Fig. 3). For instance, for L_inter-MM_ equal to 20 mm and θ = 45°, the orientation ${e}_{ct}$ proved ordered with respect to the sensing area, i.e., 2.08°, 2.16°, and 4.10° for *S*_*∞*_, *S*_*4*_, *S*_*1*_, respectively; for θ = 27° instead, the ${e}_{ct}$ demonstrated scattered, i.e., 21.28°, 7.88°, and 16.03° for *S*_*∞*_, *S*_*4*_, *S*_*1*_, respectively. Notably, results relative to N35 and N52 grade magnets (not shown) closely matched with those obtained with N45 grade magnets (differences were always below 1%), for all tested conditions.

These results suggested the existence of geometric configurations that ensured monotonic (non-random) performance across sensing surfaces, position errors below 1 mm and orientation errors around 9°, independently of the dimension of the sensing area (Fig. 3). Interestingly, these values corresponded to 10% the trajectory length and the right angle.

### Control tests

**Figure 4:**
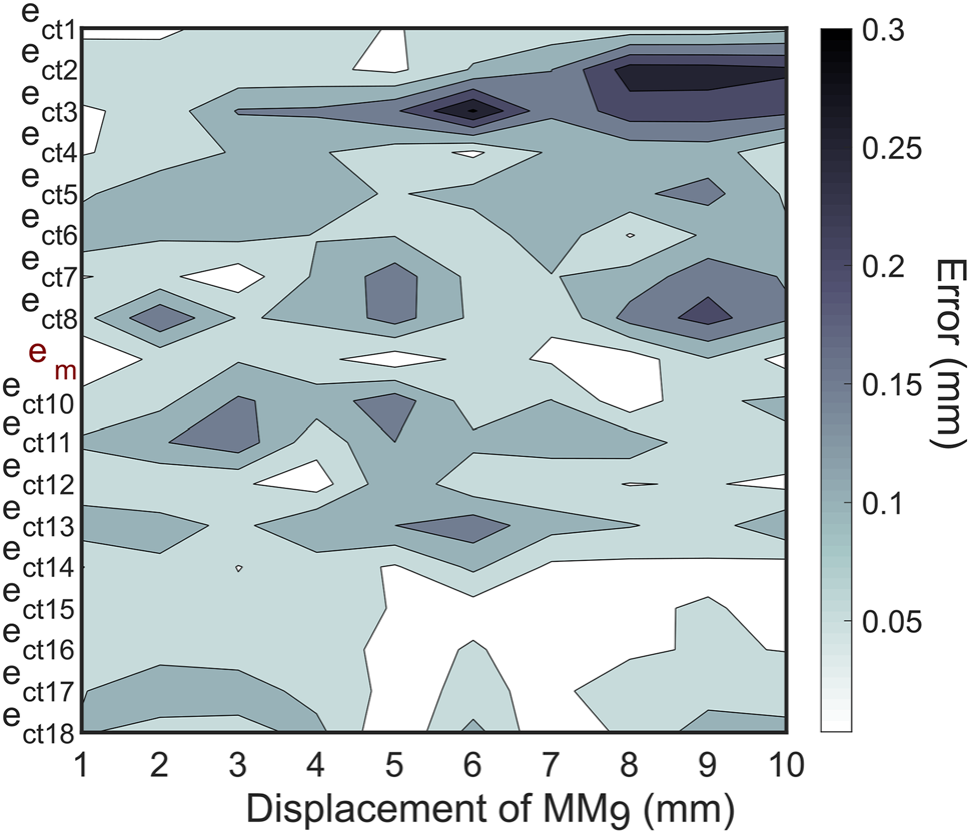
Contour plot showing the position displacement error matrix for 18 MMs (θ = 51°). The localizer was able to accurately retrieve the position of the moving magnet (i.e., MM_9_) with a maximum ${e}_{m}$ of 0.1 mm. Pointwise ${e}_{ct}$ for all non-moving MMs proved always lower than 0.31 mm.

To validate the outcomes of the simulated setups, two control tests were performed. First, one of the geometric configurations ensuring optimal performance (specifically: L_inter-MM_ = 25 mm; L_MM-sensor_ = 20 mm; θ = 51°; *S*_*∞*_) was used to assess and compare the localizer performance with 18 MMs. We found that the performance obtained while tracking 9 MMs held true and remarkably good when the number of MMs was doubled. The localizer was able to track the displacement of the moving magnet (i.e., MM_9_) along its trajectory with a maximum ${e}_{m}$ of 0.1 mm (Fig. 4). The regression line between the computed and the real displacement proved highly linear (R^2^ > 0.99, p < 0.001). ${e}_{ct}$ proved lower than 0.31 mm across all the non-moving magnets. The orientation errors exhibited similar outcomes (${e}_{m}$ lower than 1.37° and ${e}_{ct}$ lower than 1.40°). This excellent outcome suggests the possibility of achieving accurate localization of a consistent number of MMs, provided that the requirement θ ≥ 31° is met.

Secondly, results obtained in simulations were validated through an experimental setup.

**Figure 5:**
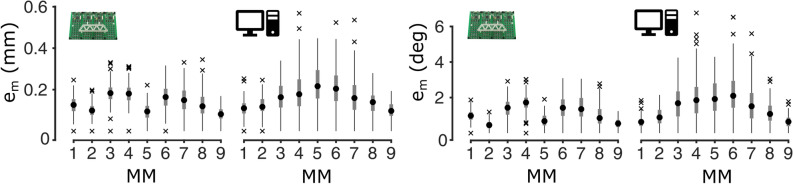
Displacement errors for both experimental (left) and simulated (right) data in the control test (θ = 51°). The crosses indicate the outliers, the boxplot encloses data within the interquartile range, the black dot is the median value, while the whiskers extend to the limits of the distribution. Results obtained with the two systems were comparable. Maximum median position (orientation) errors of 0.18 mm (1.72°) and of 0.22 mm (2.01°) were obtained for the experimental and the simulated setup, respectively.

The magnetic field of nine magnets, kept still in a pre-defined position, was sampled through 128 sensors distributed on four boards. Due to physical constraints related to the size of the acquisition system used, the MMs were arranged in a zigzag pattern, corresponding to θ = 51° (L_inter-MM_ = 25 mm; L_MM-sensor_ = 20 mm) (Fig. 1c). For comparison with the experimentally recorded data, the same configuration was reproduced in simulation, and random Gaussian noise (standard deviation of 0.04 G) was added to the recorded magnetic field. The results achieved with the magnets arranged in a linear configuration proved generalizable. In particular, with the zig-zag pattern, the localization accuracy, achieved with simulated and experimentally recorded data, proved comparable and again very good (Fig. 5). In terms of position (orientation) error, the maximum median value was 0.22 mm (2.01°) for the simulated data and 0.18 mm (1.72°) for the experimental data (N = 100 in both cases).

## Discussion

We systematically analysed the effects of the (i) magnetization grade, (ii) number of sensors, (iii) L_inter-MM_ and (iv) L_MM-sensor_ on the accuracy of a multi-magnet localizer, in a planar, simulated configuration, and we verified the validity of the simulations comparing the outcomes with those from equivalent physical systems. Overall, our results confirm the sparse information found in the literature, i.e., that larger compound/cumulative errors are obtained as L_MM-sensor_ increases^6,9,14,17,18^, and L_inter-MM_ decreases ^14^. Nonetheless they also contribute to the state of the art by suggesting geometrical relationships of the inter-magnet distance and the distance to the sensor plane, which, if respected, ensure an accurate localization of an indefinitely high number of MMs. This held true regardless of the magnetization grade of the magnets.

In agreement with our previous study^14^, we attributed the drop in accuracy to the lower θ, or R (defined as L_inter-MM_ / L_MM-sensor_). Indeed, decreasing L_inter-MM_ (or increasing L_MM-sensor_) causes the MMs to appear to the localizer as a single magnetic dipole, with a magnetic moment equal to the sum of the magnetic moments. This effect can be foreseen by observing the magnetic field distribution and its gradient (Fig. 2a,b, lower panel), which for small θ (in the example θ = 27°), do not display clearly distinguishable peaks that identify each individual magnet. This smoothening effect can be counteracted if L_MM-sensor_ decreases (or L_inter-MM_ increases) as well (Fig. 2a,b, upper panel), and our results suggest the existence of a critical ratio, namely R = 0.6 or θ =  ~ 31°, above which the shape of the field is descriptive of the individual magnets and their localization proves accurate (Fig. 3). This relationship between the shape of the field and the corresponding accuracy of the localizer is likely due to the Levenberg–Marquardt algorithm, which is in fact based on the computation of the gradient of the field, i.e., its spatial distribution. We also argue that the low sensibility of the localization accuracy w.r.t. the magnetization grade, is likely due to M being just a scaling factor in Eq. (1), thus not affecting the field/gradient distribution shape (Fig. 2a). This argument suggests that the condition of θ >  ~ 31° to achieve good accuracy, is a general rule that applies for any magnetization grade within the range of commonly available ones. On the other hand, a higher magnetization grade or magnet size could help in those situations where the signal to noise ratio is low (i.e. large noise or large L_MM-sensor_).

Furthermore, we suspect that the absence of peaks (or, in other words, the poor/filtered content) in the field spatial distribution for θ below 31° explains the lack of correlation (ρ_s_) between the errors and the sensing areas. Generally speaking, with numerical methods, the higher the number of equations (and thus, in this case, the number of sensors) the more accurate the solution^14,17–19^. However, if the gradient is not informative enough to discriminate the different magnets, the algorithm can hardly solve the inverse problem and hence converge to an optimal solution, regardless of the number of observations (or sensors). On the contrary, when the requirements on the geometry are met (θ ≥ 31°), the field distribution becomes more informative and a higher number of equations (larger sensing areas) yields to a better accuracy (Fig. 3). Notably, one should always consider that θ will vary following the MMs displacement. In particular, L_inter-MM_ could decrease and/or L_MM-sensor_ could increase, leading to smaller θ. Thus, one should always aim at maximizing θ, checking that the geometrical requirements are met irrespectively of the MMs distribution in a particular moment, and not only for the initial placement. This could be done by estimating an expected displacement of the MMs and simulating several spatial configurations^20^. Notably, the aim of the study was to provide practical guidelines to spatially distribute magnets and sensors. For this reason, we focused on the quantitative geometrical measure θ and discussed only qualitatively its relationship with the shape of the magnetic field (and its gradient). We defer to future studies to investigate which properties/features of the magnetic field affect the performance of a localization system the most.

We observed that errors generally increased with the decrease of θ. However, such monotonic trend was not always respected (Fig. 3), as already observed in our previous work^15^. This proved especially true for L_inter-MM_ = 25 mm (Fig. 3). Indeed, θ = 68° gave larger errors compared to θ = 51°. This could be due to different causes; for instance the dipole model could be less accurate in representing the MMs field for θ = 68°, as it corresponds to a minimum L_MM-sensor_ of 10 mm. At the same time, numerical approximations introduced by the finite element simulation could have a greater effect on the computation of close fields (when MMs are closer to the sensors), also considering that the latter decreases with the third power of the distance. The absence of correlation between the errors and the sensing areas in just ~ 5% of the tested configurations (θ = 56° / L_inter-MM_ = 15 mm and θ = 32° / L_inter-MM_ = 25 mm) could be explained in a similar manner. In both cases, differences among errors for different sensing areas were considerably small (maximum difference lower that 0.01 mm for θ = 56°, and equal to 0.11 mm for θ = 32°), thus numerical approximations could have had a non-negligible effect on the results.

Although ${e}_{m}$ and ${e}_{ct}$ were computed only for the specific case in which the central magnet was moved while the others were still, we argue that the outcomes are generalizable to all cases in which another MM is moving. Indeed, our results suggest that the localization of a magnet is in fact correlated to the degree of distinguishability of its peaks in the gradient, which per se is not directly correlated to which specific magnet is moving. Thus, given the same MMs arrangement, for θ ≥ 31° the distinguishability of their peaks would probably not change if a magnet other than the central one was moved. However, this remains an hypothesis that invites further studies.

A considerably large number of sensors was used for all the different sensing areas; indeed, the smallest one (*S*_*1*_) still counted 301 sensors (Table 1). This is an undesirable aspect, since it is well known that the computation time needed for the localization increases with the number of sensors^14,19^. Nevertheless, the control test with the experimental data, proved that comparable accuracies to those achieved with the simulated setups can be obtained with only 128 sensors, i.e., less than 50% of those used in S_1_. This suggests that accurate localization can be achieved with much less sensors than those used in this study. It is worth noting that, despite the exact same setup used in simulations could not be experimentally reproduced due to physical constraints, the control test carried out with experimental data allowed to further generalize the outcomes of this work. Indeed, the good repeatability of the localization observed with the physical system suggests that the rule on θ is effective also for MMs arranged on a pattern other than a line.

The present study was indeed limited in some respects. First, a planar distribution was used for both the sensors and the MMs. Thus, the validity of the outcomes of the present study in every multi-magnet tracking application should be confirmed by taking into account the specific workspace constraints. This is particularly important for biomedical applications, where the available workspace can be extremely complex as it is usually defined by anatomical structures. For instance, considering the myokinetic control interface, where a minimum L_MM-sensor_ of ~ 10 mm is desirable to avoid sensors saturation, a minimum L_inter-MM_ of 6 mm would be needed to have θ >  ~ 31°. As these distances fit smoothly to the anatomy of the forearm, we deem that such a configuration could be easily achieved and that several magnets could be implanted. Nevertheless, the conclusions drawn about the field distribution and its gradient shape suggest that comparable performance could be obtained in a more complex, anatomically relevant workspace as long as the requirements on the geometry are met (i.e., θ ≥ 31°). Secondly, in order to limit the number of combinations tested, as well as to have a better control on the parameters under study (i.e., *B*_*r*_, L_MM-sensor_, L_inter-MM_ and sensing area), the orientation of the MMs was kept fixed. This prevents us from drawing conclusions on the magnitude of the orientation error we would obtain when MMs are left free to rotate. Thirdly, the effects of external noise or sensor sensitivity were not considered. This allowed to keep the results general (i.e. not specific for a certain hardware/environment). In addition, we already observed that considering these effects in a simulated environment does not substantially alter the accuracy of a localization system^14^. Thus, we can reasonably expect the derived rules to remain valid also if external noise is considered. Nevertheless, this aspect is left to be assessed more thoroughly in future studies. Finally, the control test with the experimental setup was carried out by considering a static configuration. This may be perceived as a limitation, as it did not allow to compute ${e}_{ct}$, but just ${e}_{m}$ for all MMs. However, we can consider all the tests presented in this study as basically static, since the localizer retrieved the poses of MMs fixed in a certain position; moving one or more MMs would have only generated different static configurations. In addition, the magnitude of the errors retrieved from the experimental and the simulated setup strongly matched, with a maximum difference in ${e}_{m}$ of 0.13 mm in terms of position and of 1.32° in terms of orientation. Thus, it stands to reason that the comparability of the results would not be significantly affected when moving a MM (i.e., when considering the cross-talk component of the error). On the other hand, it remains to be tested if the dynamic performance of the localizer (i.e. the ability to retrieve the position of moving MMs) would differ consistently from the present results.

The outcomes of this work pave the way towards the development of an optimized multi-magnet localizer that could be used to track an indefinitely high number of MMs. They should serve as guidelines for the implementation of magnetic tracking systems which use is generalizable to several applications in the biomedical field. In the specific case of the myokinetic control interface, these results could be exploited to achieve a physiologically appropriate and accurate control over multiple DoFs in the prosthesis. Alternative approaches in which the measure of muscle displacements is used as a control signal for the prosthesis were proposed, such as Residual Kinetic Imaging^21^, lately referred to as Force Myography^22^, or sonomyography^23^. However, the solution here proposed holds the potential of bringing many advantages in several respects. For instance, having a magnet implanted in each target muscle would provide highly selective control signals compared to surface measurements, like those available in Force Myography^22^. The relatively small dimensions of the external sensor boards and control electronics, envisioned to be fully integrated into the socket^16^, would make the system convenient and easy-to-wear when compared, for example, with ultrasound probes used in sonography^23^. Future clinical trials will allow to quantitatively and qualitatively assess such advantages and to make a more thorough comparison with state-of-the-art solutions.

## Materials and methods

### Mathematical framework

We used the point dipole model approximation in order to simplify the solution of the localization problem (inverse problem of magnetostatics) akin to our previous studies^8,14,16^. This model approximates each MM as a point magnetic dipole located at its centre. Thus, the magnetic field B_i_ = B(x_i_), generated at the location x_i_ by a number of n dipoles, located at x_j_, j = 1, …, n, with magnetic moment equal to M $\widehat{{m}_{j}}$ (here M and $\widehat{{m}_{j}}$ are the magnitude and direction of the magnetic moment of the j-th MM) can be computed as:

1 $${{\varvec{B}}}_{i}={\varvec{B}}({x}_{i})=\sum_{j=1}^{n}\frac{{M}_{j}{\mu }_{r}{\mu }_{0}}{4\pi }\left(\frac{3\left(\widehat{{{\varvec{m}}}_{{\varvec{j}}}}\cdot {{\varvec{x}}}_{ij}\right){{\varvec{x}}}_{ij}}{{\left|{{\varvec{x}}}_{ij}\right|}^{5}}-\frac{\widehat{{{\varvec{m}}}_{{\varvec{j}}}}}{{\left|{{\varvec{x}}}_{ij}\right|}^{3}}\right), \mathrm{i}=1,\dots ,\mathrm{N}$$

where ${x}_{ij}= {x}_{i}- {x}_{i}$ and ${x}_{i}$ represent the locations of N sites (sensors) where the magnetic field is measured. The point dipole model approximation is excellent in the ideal case of infinite distance between sensors and sources (far field) while it loses accuracy when this distance becomes smaller^24^. Nevertheless, it proved sufficiently accurate in several non-ideal cases^5,14,18,25,26^. Measuring the compound magnetic field generated by the n MMs using multiple sensors allows to solve Eq. (1) in favour of ${x}_{ij}$, providing the solution to the localization problem and thus the input data required for the myokinetic control interface. However, as there is no closed form solution, the latter can only be obtained by numerical approximation. In addition, for a myokinetic control interface, it is the displacement of the MM from an offset pose (i.e., an offset position and orientation recorded with uncontracted, relaxed muscles) that reveals the degree of contraction of the muscle it is implanted in, rather than the absolute pose itself^14^.

In this work we used a Finite Element software package (Comsol Multiphysics 5.2, COMSOL Inc., Stockholm, Sweden, available at: https://www.comsol.eu/) to simulate the magnetic field produced by the MMs. The field was then sampled at N locations, and fed to a Matlab script (R2019a, MathWorks, Natick, MA) that ran the Levenberg–Marquardt algorithm^22^. Even if the orientation of the MMs did not change during the simulations (see next section), the Levenberg–Marquardt algorithm was required to retrieve both the position and orientation of the MMs (i.e. their pose) as the latter could be an important parameter to estimate/control depending on the application. All simulations were run on a desktop PC with an Intel i7-6700 CPU running at 3.4 GHz, 32 GB of RAM and Windows 7.

### Simulation setup

The simulated setup consisted of nine equidistant MMs (MM_1_-MM_9_, at L_inter-MM_ millimetres one from another) aligned at a distance L_MM-sensor_ from a sensing surface/plane (Fig. 1a). The MMs were modelled as Nd-Fe-B disc magnets (*r* = *h* = 2 mm) with their magnetization vector pointing towards the sensing plane, or the positive z direction (Fig. 1a). Three magnet grades, with a *B*_*r*_ spanning across the range of common magnetization values, were simulated, i.e. 1.12 T (N35), 1.27 T (N45) and 1.36 T (N52—maximum grade physically allowed^27^). To investigate the effects of the geometry on the localization accuracy, in a general yet realistic manner, the sensing plane included sensors uniformly distributed on a planar grid, with an inter-sensor distance (L_inter-sensor_) of 5 mm. The latter was chosen as it is roughly the size, thus the minimum distance allowed by commercial three-axis magnetic field sensors^14^.

We simulated the movements of the central MM (i.e., MM_5_) along a 10 mm long linear trajectory, perpendicular to the line of the MMs and parallel to the sensing surface (Fig. 1a,b). Such length was deemed physiologically plausible^28^. The movement of MM_5_ was simulated by assigning it static positions on 11 equidistant checkpoints along the trajectory (1 mm steps). The other MMs were kept fixed in the initial (rest) position. The orientation was kept fixed at all checkpoints for all MMs. At each step the (compound) magnetic field in the workspace was computed and stored for offline localization of the MMs, akin to our previous work^14^. The correlation between the estimated and real displacement along the trajectory was quantified through the coefficient of determination (R^2^) and associated *p* value. The *p* value was computed by transforming the correlation to create a t statistic having X-2 degrees of freedom, where X is the number of checkpoints.

In order to identify the ideal localization accuracy (our benchmark) we simulated a practically infinite plane of sensors (condition *S*_*∞*_). This was achieved by truncating the plane where the sensors in the grid measured an almost null magnetic field, and in practice meant a plane being 165 mm larger than the area covered by the magnets along the x axis (and 55 mm larger on the y axis) for a total of 2889 sensing sites (Fig. 1b). We then analysed the effects of reducing the sensing surface on the localization accuracy by incrementally removing the data acquired by the sensors at the edges, for a total of six sensing surfaces (i.e., *S*_*∞*_*, S*_*5*_*-S*_*1*_). In particular, the distance between the edge of the plane and the external magnets was reduced from 165 to 5 mm, along the x direction, and from 55 to 5 mm along the y axis (Table 1). We expected the error to increase in accordance with the reduction of the sensing surface.

Finally, in order to gather insights on the effects of the geometry on the localization accuracy, the simulations were repeated for different combinations of L_inter-MM_ and L_MM-sensor_ building from the knowledge gained in our previous study^14^. Specifically, the L_inter-MM_ ranged between 15 and 25 mm, in 5 mm steps whereas L_MM-sensor_ varied from 10 to 40 mm in 10 mm steps. Such distances, and in particular their ratio R = L_inter-MM_ / L_MM-sensor_, was used to define θ (Fig. 1a), through the following expression:

2 $$\theta ={\mathrm{tan}}^{-1}\left(R\right).$$

θ served as a compact geometric descriptor to cluster and analyse the outcomes of the study across the several assessed conditions.

### Control tests

One of the geometric configurations that ensured optimal performance was assessed in terms of its localization accuracy with an increased number of MMs, in order to verify whether the rules found could generalize with many more magnetic objectives. In particular, we simulated a setup including 18 MMs (N45 grade), considering that this is the number of the extrinsic muscles of the hand. Like previous simulations, the MMs were aligned on a line parallel to the sensors plane, and the one occupying the central position, i.e. MM_9_, was moved along a 10 mm pointwise trajectory. The simulation protocol was consistent with that used for nine magnets.

As a further control, we compared the localization accuracy achieved with simulated and experimentally recorded data, using the same (optimal) combination of L_inter-MM_ and L_MM-sensor_ identified and used with 18 MMs (Fig. 1c). Four custom boards each one including 32 three-axis magnetic field sensors (MAG3110, Freescale Semiconductor Inc., full-scale output of ± 10 G and sensitivity of 1 mG) were aligned and used to collect the magnetic field generated by nine MMs (N45 grade). Sensors on each board were arranged in a 4 × 8 matrix, with a L_inter-sensor_ of 9 mm inside each matrix, and a L_inter-sensor_ of ~ 17 mm between adjacent matrices (i.e., adjacent boards). MMs were placed over the sensor plane, supported by a frame parallel to the sensors (Fig. 1c), having the magnetization vectors perpendicular to the sensor surface. In particular, given that the geometric configuration that ensured optimal performance in simulations proved physically larger than the boards, the MMs were arranged in an equilateral zigzag pattern having the side length of L_inter-MM_ (Fig. 1c). The MMs were kept steady in their positions for the whole duration of the test and 100 readings were recorded. The same physical setup was reproduced in simulation. Random Gaussian noise was added on the sampled magnetic field (100 repetitions) in order to simulate the effects of measurement uncertainties by mimicking the non-ideal accuracy and repeatability of real sensors. The Gaussian noise had a standard deviation of 0.004 G, corresponding to the noise characteristics measured on a commercial magnetometer (MAG3110, Freescale Semiconductor, Inc.).

The localization errors were computed for all the 100 acquisitions of the experimental and simulated setup and were assessed relatively to the localized pose corresponding to the first sample. The aim was to investigate the transferability of the outcomes obtained in a simulated environment into the real world.

### Performance metrics

The accuracy of the multi-magnet localizer was assessed in terms of its position (${E}_{d}$) and orientation (${E}_{o}$) displacement errors when localizing multiple MMs. ${E}_{d}$ was computed as the Euclidean distance between the real and the estimated displacement with respect to an initial point; similarly, ${E}_{o}$ was computed as the angular difference between the real and the estimated orientations. As in our previous work, for a system comprising *n* magnets and neglecting environmental factors, position error (${E}_{d}$) and orientation error (${E}_{o}$) at a certain pose *p*_*x*_ are given by:

3 $${\mathrm{E}}_{\mathrm{d}}\approx { e}_{{\mathrm{m}}_{\mathrm{d}}}\left({p}_{x}\right)+\sum_{j=1}^{n-1}{e}_{{\mathrm{ct}}_{\mathrm{d}} j}\left({p}_{x}\right)$$

4 $${\mathrm{E}}_{\mathrm{o}}\approx {e}_{{\mathrm{m}}_{o}}\left({p}_{x}\right)+\sum_{j=1}^{n-1}{e}_{{\mathrm{ct}}_{o }j}\left({p}_{x}\right)$$

where ${e}_{m}$, is the model error referred to the moving magnet, and ${e}_{ct}$, is the cross-talk error, which represents the false prediction of simultaneous displacement on the non-moving magnets.

For the simulated setups (Fig. 1a,b) we computed the position and orientation displacement errors as the 95^th^ percentile of the errors along the trajectory (i.e. in a very conservative manner)^14^. For the comparison between simulation and recorded data (Fig. 1c), we computed only ${e}_{m}$ since the magnets were still and thus no cross-talk interference was present.

## Data Availability

All data are available in the main text or cited resources mentioned in the text.

## References

[1] Cleary K, Peters TM (2010). Image-guided interventions: technology review and clinical applications. Annu. Rev. Biomed. Eng..

[2] Franz AM (2014). Electromagnetic tracking in medicine—a review of technology, validation, and applications. IEEE Trans. Med. Imaging.

[3] Hu C (2016). Locating intra-body capsule object by three-magnet sensing system. IEEE Sens. J..

[4] Salerno M (2012). A discrete-time localization method for capsule endoscopy based on on-board magnetic sensing. Meas. Sci. Technol..

[5] Pham DM, Aziz SM (2014). A real-time localization system for an endoscopic capsule using magnetic sensors. Sensors (Switzerland).

[6] Maréchal L (2016). Design optimization of a magnetic field-based localization device for enhanced ventriculostomy. J. Med. Devices Trans. ASME.

[7] Krueger S, Timinger H, Grewer R, Borgert J (2005). Modality-integrated magnetic catheter tracking for x-ray vascular interventions. Phys. Med. Biol..

[8] Tarantino S, Clemente F, Barone D, Controzzi M, Cipriani C (2017). The myokinetic control interface: tracking implanted magnets as a means for prosthetic control. Sci. Rep..

[9] Rouse EJ, Nahlik DC, Peshkin MA, Kuiken TA (2011). Development of a model Osseo-Magnetic Link for intuitive rotational control of upper-limb prostheses. IEEE Trans. Neural Syst. Rehabil. Eng..

[10] Hu C, Meng MQH, Mandal M (2005). Efficient linear algorithm for magnetic localization and orientation in capsule endoscopy. Annu. Int. Conf. IEEE Eng. Med. Biol. Proc..

[11] Sherman JT, Lubkert JK, Popovic RS, Disilvestro MR (2007). Characterization of a novel magnetic tracking system. IEEE Trans. Magn..

[12] Yang W, Hu C, Li M, Meng MQH, Song S (2010). A new tracking system for three magnetic objectives. IEEE Trans. Magn..

[13] Taylor CR, Abramson HG, Herr HM (2019). Low-latency tracking of multiple permanent magnets. IEEE Sens. J..

[14] Tarantino S, Clemente F, De Simone A, Cipriani C (2019). Feasibility of tracking multiple implanted magnets with a myokinetic control interface: simulation and experimental evidence based on the point dipole model. IEEE Trans. Biomed. Eng..

[15] Yabukami S (2000). Motion capture system of magnetic markers using three-axial magnetic field sensor. IEEE Trans. Magn..

[16] Clemente F, Ianniciello V, Gherardini M, Cipriani C (2019). Development of an embedded myokinetic prosthetic hand controller. Sensors.

[17] Hu, C., Ma, T. & Meng, M. Q. H. Sensor arrangement optimization of magnetic localization and orientation system. *IEEE ICIT 2007—2007 IEEE Int. Conf. Integr. Technol.* 311–315 (2007). 10.1109/ICITECHNOLOGY.2007.4290485

[18] Schlageter V, Besse PA, Popovic RS, Kucera P (2001). Tracking system with five degrees of freedom using a 2D-array of Hall sensors and a permanent magnet. Sen. Actuators, A Phys..

[19] Talcoth, O. & Rylander, T. *Optimization of sensor positions in magnetic tracking*. Chalmers University of Technology, Göteborg, Sweden, Technical Report (2011).

[20] Milici S (2020). The myokinetic control interface: how many magnets can be implanted in an amputated forearm? Evidence from a simulated environment. IEEE Trans. Neural Syst. Rehabil. Eng..

[21] Phillips SL, Craelius W (2005). Residual kinetic imaging: a versatile interface for prosthetic control. Robotica.

[22] Belyea A, Englehart K, Scheme E (2019). FMG versus EMG: a comparison of usability for real-time pattern recognition based control. IEEE Trans. Biomed. Eng..

[23] Shi J, Chang Q, Zheng YP (2010). Feasibility of controlling prosthetic hand using sonomyography signal in real time: preliminary study. J. Rehabil. Res. Dev..

[24] Sadiku, M. Magnetic Forces, Materials, and Devices. In *Elements of Electromagnetics,* 304–369 (Oxford University Press, 2000).

[25] Wang, X., Meng, M. Q. H. & Hu, C. A localization method using 3-axis magnetoresistive sensors for tracking of capsule endoscope. In *Annual International Conference of the IEEE Engineering in Medicine and Biology—Proceedings,* 2522–2525 (2006). 10.1109/IEMBS.2006.260711 10.1109/IEMBS.2006.260711 17946518

[26] Andrä W (2000). A novel method for real-time magnetic marker monitoring in the gastrointestinal tract. Phys. Med. Biol..

[27] Du, B. ( 12 ) Patent Application Publication ( 10 ) Pub . No .: US 2010 / 0035098 A1 Patent Application Publication. **1**, (2010).

[28] Chao, E. Y. Hand joint orientation and range of motion. In *Biomechanics of the Hand: a basic research study,* 73–96 (World Scientific, 1989).

